# Genetic Analysis and Fine Mapping of Spontaneously Mutated Male Sterility Gene in Chinese Cabbage (*Brassica rapa* L. ssp. *pekinensis*)

**DOI:** 10.3390/plants14050779

**Published:** 2025-03-03

**Authors:** Qian Xu, Xiaochun Wei, Yanyan Zhao, Jianqi Feng, Peiyun Wang, Cong Ding, Wenjing Zhang, Henan Su, Weiwei Chen, Fang Wei, Yuxiang Yuan, Xiaowei Zhang

**Affiliations:** 1 Institute of Vegetables, Henan Academy of Agricultural Sciences, Graduate T&R Base of Zhengzhou University, Zhengzhou 450002, China; 16637656206@163.com (Q.X.); jweixiaochun@126.com (X.W.); zhaoyanyan9621@163.com (Y.Z.); zhangwj050@163.com (W.Z.); 18810835083@163.com (H.S.); weiwei_chen15134@zzu.edu.cn (W.C.); fangwei@zzu.edu.cn (F.W.); 2 School of Agricultural Sciences, Zhengzhou University, Zhengzhou 450001, China; 3 Kaifeng Academy of Agriculture and Forestry Sciences, Kaifeng 475000, China; ks6918@163.com (J.F.); fengjq18567@163.com (P.W.); d_cong123@163.com (C.D.)

**Keywords:** Chinese cabbage, genic male sterility, map-based cloning, acyl-CoA synthetase 5, phenylpropanoid biosynthesis

## Abstract

Chinese cabbage (*Brassica rapa* L. ssp. *pekinensis*), an important traditional vegetable indigenous to China, is a typical cross-pollinated Brassica crop exhibiting pronounced heterosis. However, its small flower organs make artificial pollination for hybrid seed production highly challenging. The use of male-sterile lines has emerged as a crucial approach in hybrid seed production. Therefore, understanding the genetic and molecular mechanisms underlying male sterility in Chinese cabbage holds profound theoretical and economic importance and is pivotal for advancing Chinese cabbage crossbreeding. Here, cytological comparative analysis of anthers from sterile line 366-2S and fertile line 366-2F revealed abnormalities in 366-2S during the late tetrad stage, including delayed tapetum degradation and the aggregation of tetrad microspores without separation, which prevented pollen production and caused male sterility. Construction of the F_2_ segregating population, with 366-2S as the female parent and genetically diverse fertile material Y636-9 as the male parent, indicated that male sterility in 366-2S is controlled by a single recessive gene. Using bulked segregant analysis sequencing and kompetitive allele-specific polymerase chain reaction (KASP) technology, the sterile gene was mapped to 65 kb between the PA11 and PA13 markers, with 11 genes in the candidate region. Functional annotation, expression, and sequence variation analyses identified *BraA09g012710.3C*, encoding acyl-CoA synthetase 5, as a candidate gene for 366-2S male sterility. Quantitative real-time polymerase chain reaction analysis revealed minimal expression of *BraA09g012710.3C* in 366-2S but high expression in the flower buds of 366-2F. Further analysis of candidate gene DNA sequences identified a large deletion encompassing *BraA09g012710.3C*, *BraA09g012720.3C*, *BraA09g012730.3C*, and *BraA09g012740.3C* in sterile line 366-2S (A09: 7452347–7479709). Cloning and verification of the other three deleted genes in the F_2_ population via agarose gel electrophoresis confirmed their presence in F_2_ sterile individuals, indicating that their deletion was not associated with male sterility, underscoring *BraA09g012710.3C* as the key gene driving male sterility in 366-2S.

## 1. Introduction

Male sterility in plants refers to the condition in which plant anthers fail to undergo normal cracking or produce inactive pollen and male gametes. This phenomenon was first observed by the German botanist Joseph Gottlieb Kölreuter in 1763 [1]. In nature, plant male sterility manifests in various forms. Plant male sterility can be categorized into two types based on genotype: genic male sterility (GMS) and cytoplasmic male sterility (CMS) [2]. Studies have indicated that CMS lines may suffer from issues such as reduced genetic diversity, increased susceptibility to diseases, and unstable fertility restoration. These challenges can be addressed through the use of GMS lines [3,4].

In agriculture, hybrid development is widely practiced to harness heterosis, especially in vegetables. Artificial emasculation is typically employed during seed production to facilitate improved breeding [5]. Chinese cabbage (*Brassica rapa* L. ssp. *pekinensis*), which is known for its strong heterosis, is a typical cross-pollinated crop [6]. Male sterility circumvents breeding challenges such as labor-intensive emasculation and hand pollination while preventing undesired self-pollination or inbreeding. In addition, male sterility provides an advantage in F_1_ hybrid seed production [7]. GMS was first reported in cabbage [8] and cauliflower [9]. GMS is controlled by nuclear genes typically governed by single recessive alleles and is prevalent among angiosperms [10]. Most reported male-sterile mutants arise spontaneously [10]. GMS generally follows Mendelian inheritance [10,11], with most spontaneous or induced mutations being controlled by a single recessive gene and occasionally by multiple alleles [12,13].

Map-based cloning, a gene-mapping technology using molecular markers, has evolved significantly. Typically, starting from the mutant phenotype, markers such as simple sequence repeats (SSRs) and insertions and deletions (Indels) are used to narrow down the mapping interval until the mutant gene is identified [14]. Despite its complexity in marker screening, this technique has been streamlined with the advent of high-throughput sequencing technology. Bulked segregant analysis (BSA) combined with kompetitive allele-specific polymerase chain reaction (KASP) technology now facilitates the rapid and efficient localization of target genes [15], thereby aiding plant breeders in overcoming traditional breeding challenges [16].

In Chinese cabbage, nuclear male sterility primarily exhibits single recessive or multiple allelic genetic types, and several genes linked to nuclear male sterility have been identified. Numerous genes associated with male sterility have been identified in Brassica crops. For example, ‘Aijiaohuang’ Chinese cabbage (*B. campestris* ssp.) developed a male-sterile AB line Bcajh97-01A/B controlled by a single recessive mutation, with differences in pollen formation found between ‘Bcajh97-01A’ and ‘Bcajh97-01B’ [17]. Pollen abortion in these lines is caused by early meiotic cytoplasmic division during pollen development [18,19]. Amplified fragment length polymorphism (AFLP) markers linked closely to the *GMS* gene were identified by Ying et al., with a distance of <1 cM [20]. Wei et al. identified AFLP and sequence-characterized amplified region (SCAR) markers closely linked to the *MS* gene, with the AFLP01 (SCAR01) and AFLP04 markers located at 2.3 cM and 7.8 cM of the MS locus, respectively [21].

In recent years, the majority of male-sterile mutants in Chinese cabbage have been induced through mutagenesis. For example, Zhao et al. identified a recessive nuclear gene controlling male sterility in the *ftms* mutant, with MutMap and KASP analysis suggesting *BrGGL7* (*BraA05g022470.3C*), encoding a Gly-Asp-Ser-Leu (GDSL) esterase/lipase, as a potential mutant gene candidate [22]. Dong et al. discovered three allelic male-sterile mutants (msm2-1/2/3) in mutagenized Chinese cabbage offspring, all controlled by the same gene, with MutMap and KASP identifying *BraA10g019050.3C*, homologous to *AtMS1*, as a candidate target gene encoding a Plant homeodomain (PHD)-finger transcription factor regulating pollen development [23]. Zou et al. isolated three allelic male-sterile mutants (msm1-1, msm1-2, and msm1-3) from an ethyl methane sulfonate (EMS)-induced ’FT’ Chinese cabbage double haploid (DH) line, and MutMap and KASP identified different single-nucleotide polymorphisms (SNPs) associated with msm1-1/2/3 male sterility [24].

In the present study, a recessive nuclear male-sterile mutant, 366-2S, was identified in a natural population of Chinese cabbage. Phenotypic and cytological observations revealed severe deficiencies in pollen wall formation, delayed degradation of the tapetum layer, and thickened callase layers impeding microspore development, leading to microspore abortion. F_2_ populations, integrating genetically diverse Y636-9 and 366-2S, were subjected to GMS gene mapping using BSA and KASP methods. Sequence analysis of the mapped 65 kb interval highlighted four gene deletions, including *BraA09g012710.3C*, a homolog of Arabidopsis *ACOS5*, which is specifically expressed in anthers and enriched in the phenylpropanoid metabolic pathway. Abnormalities in the phenylpropanoid metabolic pathway affect the development of the tapetum layer, which is a crucial source of nutrients, raw materials, and energy for pollen formation and secretes callase enzymes that are vital for microspore release during the tetrad period. Impaired tapetum layer development results in pollen abortion and the failure of microspore formation, leading to male sterility.

## 2. Results

### 2.1. Phenotypic Observations of the 366-2F (Fertile) and 366-2S (Sterile) Lines

**Figure 1:**
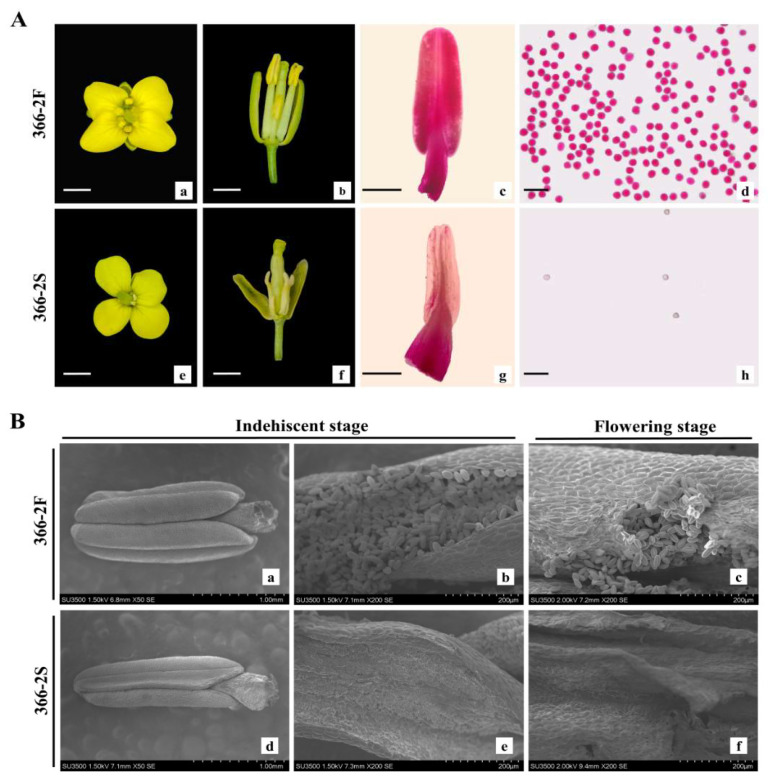
Phenotypic observations and anther scanning electron microscopy of the 366-2F (fertile) and 366-2S (sterile) lines. (**A**) (**a**,**b**): Floret at the anthesis stage, exhibiting normal flower organs, in fertile line 366-2F. (**e**,**f**): Floret at the anthesis stage, exhibiting shorter filaments and anthers without pollen, in sterile line 366-2S. (**c**,**d**): 366-2F anther rectification and pollen grain staining. (**g**,**h**): 366-2S anther rectification and pollen grain staining. (**a**,**b**,**e**,**f**): bar = 5 mm; (**c**,**g**): bar = 1 mm; and (**d**,**h**): bar = 50 µm. (**B**) (**a**): 366-2F anther at the indehiscent stage. (**b**): Anatomical diagram of 366-2F anther at the indehiscent stage. (**c**): 366-2F anther anatomy at the flowering stage. (**d**): 366-2S anther at the indehiscent stage. (**e**): Anatomical diagram of 366-2S anthers at the indehiscent stage. (**f**): 366-2S anther anatomy at the flowering stage. (**a**,**d**): bar = 1 mm; (**b**,**c**,**e**,**f**): bar = 200 μm.

Pure and heterozygous fertile lines 366-2F and heterozygous fertile lines were grown, and sterile line 366-2S was isolated and identified from the heterozygous fertile lines. A comparison of the flower organ morphology of 366-2F and 366-2S revealed distinct differences in anther morphology (Figure 1A(a,b,e,f)). The anthers of line 366-2F appeared yellow and plump (Figure 1A(b)) and were capable of normal dehiscence and pollen release, while the anthers of line 366-2S were dry, gray, and shriveled (Figure 1A(f)) and devoid of pollen grains, indicating impaired anther development. Alexander staining confirmed the absence of pollen grains and complete sterility in 366-2S (Figure 1A(g,h)) [25].

### 2.2. Anther and Pollen Characteristics of 366-2S and 366-2F

SEM revealed significant structural differences in anthers and pollen between sterile and fertile plants. Fertile anthers were larger than their sterile counterparts. During the non-dehiscent period, observations revealed small, thin anthers in 366-2S, in contrast with the plump anthers noted in 366-F (Figure 1B(a,d)). The anatomical analysis confirmed the absence of active pollen grains in 366-2S anthers, whereas 366-2F anthers contained abundant active pollen grains (Figure 1B(b,e)). At the pollen dispersal stage, the anthers of 366-2S appeared wilted and devoid of scattered pollen grains, whereas the anthers of 366-2F were covered with scattered pollen grains (Figure 1B(c,f)).

### 2.3. Microspore Development in 366-2S and 366-2F

**Figure 2:**
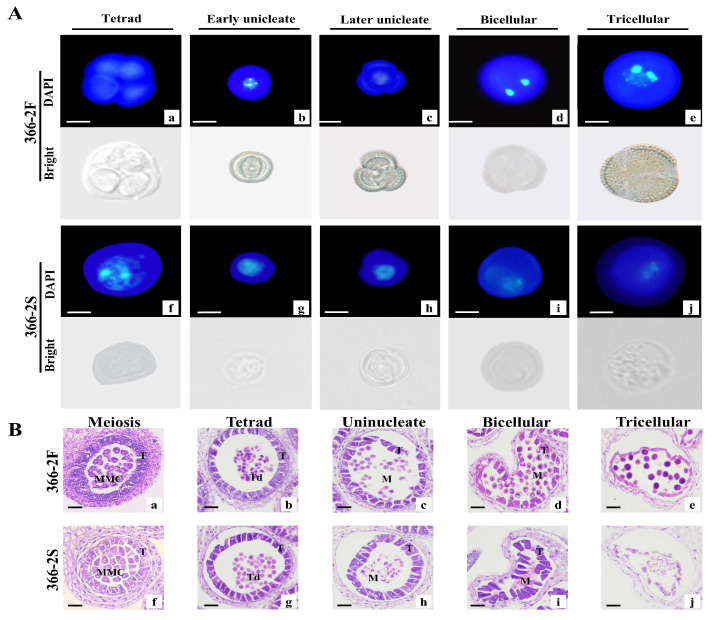
DAPI staining of pollen microspores and anther paraffin sections. (**A**). (**a**–**e**): Microspore development process of fertile line 366-2F. (**f**–**j**): Microspore development process of sterile line 366-2S. Bar = 50 µm. (**B**). (**a**–**e**): Microspore development of 366-2F pollen. (**f**–**j**): Pollen microspore development process of 366-2S, with abnormal development beginning in the tetrad stage. T: tapetum; MMC: microspore mother cell; Td: tetrad; and M: microspore. Bar = 20 µm.

Intriguingly, the anthers of 366-2S exhibited almost no fertile pollen grains at the late stage of development. To further investigate this phenomenon, DAPI-stained spores at different developmental stages were observed to determine whether the abnormal development of the microspore nucleus occurred. According to the results, the microspore development process in 366-2F showed a typical microspore development behavior pattern (Figure 2A(a–e)). Typically, during the tetrad stage, the tetraspore formed by meiosis is surrounded by thick callose. At the uninucleate stage, the callase enzyme secreted by the tapetal cells of the anther degrades the callose wall and releases the microspores in the tetrasporophyte to form a single microspore. At this time, the nucleus is located in the center of the cell, and the fluorescence is strong. At the late uninucleate stage, the nucleus gradually moves from the center to the side of the cell as the microspores continue to absorb nutrients from the tapetum cells and grow. In the binucleate stage, the single nucleus of the microspore undergoes the first mitosis to form two nuclei: a sperm nucleus and a vegetative nucleus. In the trinuclear stage, the vegetative nucleus is retained, while the sperm nucleus forms two sperm nuclei through the second mitosis. Compared with 366-2F, the microspore development of 366-2S began to show abnormalities at the tetrad stage. It could be that the pollen mother cells failed to separate, or it might be that the tetrad microspores aggregated into clusters and were unable to form individual microspores. At the early and late monokaryotic stages, 366-2S showed abnormal nuclear morphology, with lighter nuclear staining and slight dispersion. In the binucleate stage, the nuclear dispersion of 366-2S further deepened. In the trinucleate stage, the nuclei of 366-2S were barely stained and disappeared. Finally, 366-2S failed to form individual microspores (Figure 2A(f–j)).

### 2.4. Anther Paraffin Section Staining for Observation

Further analysis of anther development via Hematoxylin-eosin staining revealed no discernible differences between the 366-2F and 366-2S lines during the meiosis and tetrad stages (Figure 2B(a,b,f,g)), with a normal tapetum and microspore mother cell development. However, during the mononuclear and binuclear stages, 366-2F tetrads separated normally, releasing individual microspores (Figure 2B(c,d)), whereas 366-2S tetrads remained clustered (Figure 2B(h,i)) and failed to form individual microspores. At the trinucleate stage, 366-2F microspores were abundant and round, filling the anther locule (Figure 2B(e)), while the 366-2S anther locules were atrophied with sparse contents, it is obvious that they cannot form viable pollen [25]. Additionally, the tapetum layer in 366-2S appeared thicker during the dinuclear and trinuclear phases, which suggests that the delayed degradation of the tapetum layer may contribute to male sterility (Figure 2).

### 2.5. Genetic Analysis of Sterile Traits in 366-2S

The genetic analysis of the sterile trait in 366-2S was conducted using the F_1_ and F_2_ populations. The F_1_ plants were fertile, indicating the dominance of the fertility trait. The small F_2_ population consisted of 1448 fertile plants and 435 sterile plants, with a chi-square test ratio of 3:1 (Table S1). The larger population consisted of 5118 fertile plants and 1638 sterile plants (χ^2^ = 2.25 < χ^2^ 0.05 = 3.84). Additionally, eighteen different genetic background Double Haploid (DH) lines were crossed with 366-2S, resulting in fertile hybrid offspring (Table S2). These results suggest that the sterility trait in 366-2S is controlled by a single recessive gene.

### 2.6. Fine Mapping of the Sterile Gene

**Figure 3:**
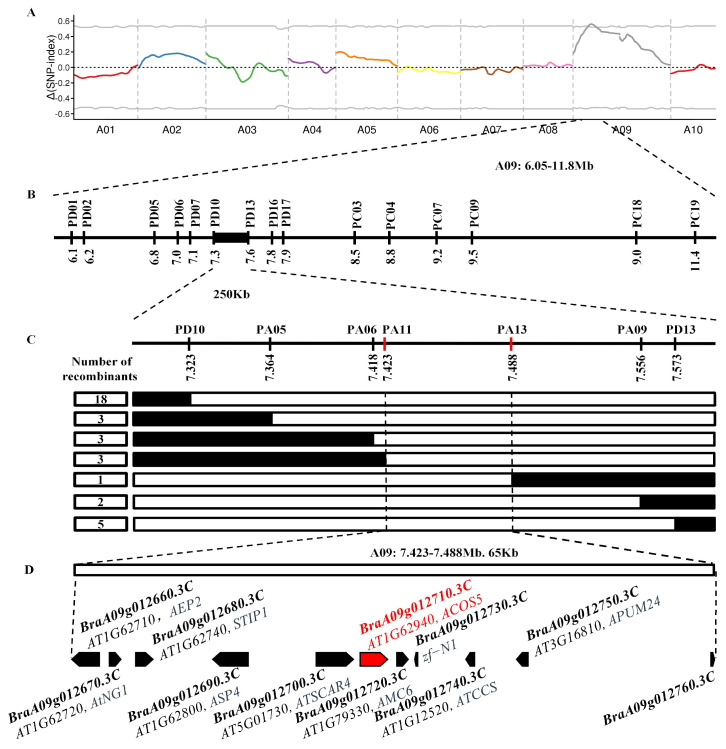
Fine mapping of the nuclear sterility gene. (**A**) Single-nucleotide polymorphism (SNP)-index analysis of fertility in the F_2_ population. (**B**) Initial physical location map of the sterility gene. (**C**) Fine mapping of the nuclear sterility gene. (**D**) Details of candidate genes in the interval.

To identify the candidate sterile gene, 30 sterile plants (366S) and 30 fertile plants (366F) were selected from the F_2_ population. Two mixed pools, one sterile and one fertile, were created for BSA-seq. In total, 95,290,539 and 56,005,553 raw reads were obtained from the 366-2S and 366-2F lines, respectively, with 963,991 SNPs and 241,679 Indels identified between the two DNA pools (Table S3). Using sliding window analysis based on Δ (SNP index), a 5.75 Mb candidate region was identified on chromosome A09 spanning from 6.05 to 11.80 Mb with a confidence level of 0.01 (Figure 3A).

Based on the BSA-seq results, 40 KASP markers were developed, with 15 showing polymorphism between the parental lines. These markers were employed to conduct genotype linkage analysis in 325 F_2_ plants. The sterile gene locus was initially mapped between the PD10 and PD13 markers on chromosome A09, covering a physical interval of 249.98 kb (Figure 3B).

Further refinement of the sterile gene locus involved screening 708 F_2_ sterile plants using the PD10 and PD13 markers, identifying 23 recombinant plants. These recombinants, along with the parental lines Y636-9 and 366-2S, were genotyped using the PA05, PA06, PA09, PA11, and PA13 markers. This analysis revealed three recombinant plants between marker PA11 and the sterile gene, while one recombinant plant was identified between marker PA13 and the sterile gene. Ultimately, the sterile gene was localized between the PA11 and PA13 markers, within a physical distance of 65 kb (Figure 3C).

### 2.7. Functional Annotation Analysis of Candidate Genes

Based on the fine mapping results, the gene sequence in the finely mapped 65 kb candidate interval was retrieved from the Brassica database (http://brassicadb.cn/, accessed on 12 April 2024). A total of 11 genes were screened in this candidate interval (Figure 3D, Table S4). Among them, *BraA09g012710.3C* encodes acyl-CoA synthetase 5. It is known that acyl-CoA synthetase is involved in lipid metabolism. Studies have demonstrated that *ACOS5* is necessary for pollen biosynthesis and pollen development in *Arabidopsis thaliana* [26]. Therefore, *BraA09g012710.3C* may be a key candidate gene for male sterility.

### 2.8. Comparison of BrA09g012710.3C Gene Sequences Between Male Fertile and Male-Sterile Plants

**Figure 4:**
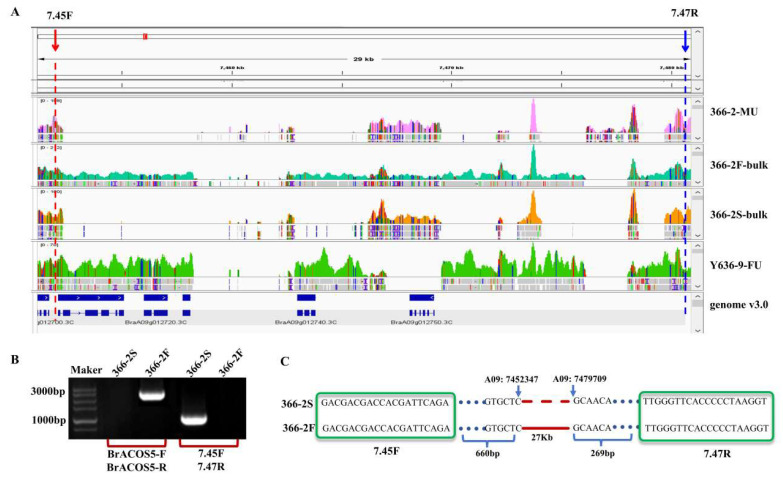
DNA sequence analysis of candidate genes. (**A**) Visualization analysis results of candidate interval sequencing reads. (**B**) Amplification results of full-length and deletion fragments of *BraA09g02710.3C* in 366-2S and 366-2F. (**C**) Missing fragment pattern. Note: 366-2-FU stands for parent material 366-2, Y636-9-MU stands for parent material Y636-9.

According to the BSA-seq sequencing results, 366-2S exhibited a large fragment deletion from the candidate gene *BraA09g012710.3C* (partial sequence) (Figure 4A). The full-length primer (Table S5) of *BraA09g012710.3C* was designed to amplify 366-2S and 366-2F via PCR. The results demonstrated that the amplified band size of 366-2F was in line with expectations (2975 bp), while 366-2S exhibited no amplified band (Figure 4B). Primers were designed before and after the deletion fragment (approximately 28 kb), and PCR amplification was performed on the 366-2S and 366-2F lines. At the limit of detection of the PCR program, if no deletions were present, the bands could not be successfully amplified because they were too long. The results indicated that 366-2S had amplified bands, while pure 366-2F had no amplified bands (Figure 4B).

**Figure 5:**
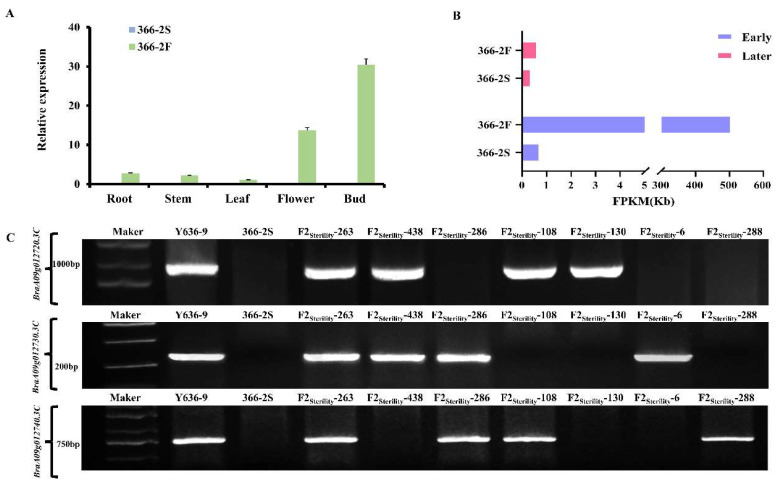
Expression level of *BraA09g012710.3C* and electrophoretic verification of three deleted genes. (**A**) Relative expression levels of *BraA09g012710.3C* in different tissues of the 366-2S and 366-2F lines. (**B**) Breakpoint histograms of 366-2S and 366-2F at the early and late FPKM values. (**C**) Results of agarose gel electrophoresis validation of the expression of *BraA09g012720.3C*, *BraA09g012730.3C*, and *BraA09g012740.3C* in F_2_ sterile individuals. Note: Y636-9 is a fertile parent; 366-2S is a sterile parent; F_2_-Sterility is the sterile F_2_ generation; and FPKM is fragments per kilobase of transcript per million mapped reads. Note: the seven numbers in the Figure, 263, 438, 286, 108, 130, 6, and 288 represent sterile single plant numbers, and these seven plants were selected by us based on different combinations of the three genes.

The PCR products of the amplified DNA regions of 366-2S underwent sequencing, and the sequencing results were compared and analyzed using DNAMAN (https://www.lynnon.com/dnaman.html, accessed on 26 February 2025). According to the results, there was a deletion of 27 kb (A09: 7452347-7479709) in 366-2S (Figure 4C), which included the deletions of the following four genes: *BraA09g012710.3C*, *BraA09g012720.3C*, *BraA09g012730.3C*, and *BraA09g012740.3C*. To determine whether the deletion of the other three genes (*BraA09g012720.3C*, *BraA09g012730.3C*, and *BraA09g012740.3C*) was related to fertility, specific primers were designed for each of the three genes (Table S6). The presence of these genes in F_2_ sterile individuals was verified, and the percentages of these genes were counted using agarose gel electrophoresis (Table S7). The results of electrophoresis showed that 366-2S plants still exhibited male sterility even when these three genes were not missing (Figure 5C). The mRNA expression indicated that *BraA09g012710.3C* was not or barely detectable in 366-2S at the early stage, while it was highly detectable in 366-2F, with a highly significant difference (Figure 5B), while *BraA09g012720.3C*, *BraA09g012730.3C*, and *BraA09g012740.3C* had low expression levels in 366-2S and 366-2F (Table S8). These results indicate that the deletion of the genes *BraA09g012720.3C*, *BraA09g012730.3C*, and *BraA09g012740.3C* did not affect fertility and that *BraA09g012710.3C* was the key gene influencing the fertility of 366-2S.

### 2.9. Analysis of Candidate Gene Expression

To further determine the candidate genes, specific quantitative primers (Table S9) were designed for *BraA09g012710.3C*, and the expression of *BraA09g012710.3C* in different tissues of sterile line 366-2S and fertile line 366-2F was detected using qPCR. The results showed that the expression of BraA09g012710.3C in the two materials was significantly different. It is almost not expressed in all organs of the sterile line 366-2S, but it is expressed in the fertile line 366-2F, especially in the buds, where the expression in the fertile material is significantly higher than that in the sterile material. Based on this, we speculate that the difference in the expression of *BraA09g012710.3C* affects the fertility of 366-2S (Figure 5A).

### 2.10. Phylogenetic Analysis of the ACOS5 Gene

**Figure 6:**
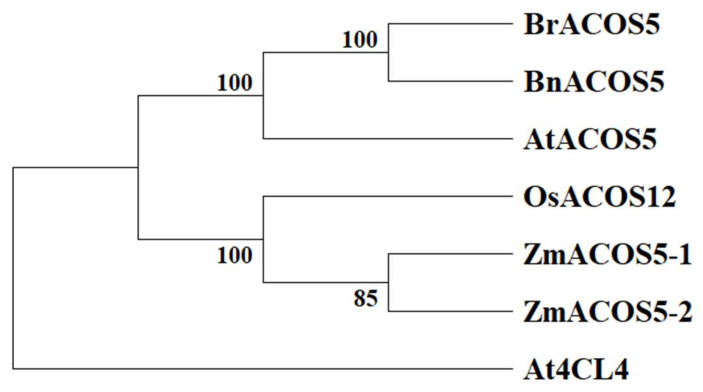
Phylogenetic tree of the BrACOS5 gene. Note: At, *Arabidopsis thaliana*; Bn, *Brassica napus*; Br, *Brassica rapa*; Zm, *Zea mays*; Os, *Oryza sativa*.

To examine the evolutionary conservation of the *ACOS5* gene, the full-length sequence of *BraA09g012710.3C* protein was searched on the National Center for Biotechnology Information (NCBI), and a BLASTP search was performed for homologous protein sequences in different species. The protein sequence of *ACOS5* in Chinese cabbage was analyzed using a phylogenetic tree with the protein sequences of maize, rice, A. thaliana, and rapeseed, the addition of *At4CL4* as an outgroup is phylogenetic tree rooting. The results revealed that *BraA09g012710.3C* had the highest similarity with *BnACOS5* and Arabidopsis *ACOS5* (Figure 6), suggesting that *BraA09g012710.3C*, like Arabidopsis *ACOS5*, may be involved in anther development.

### 2.11. Analysis of Differential mRNA Expression

The 366-2S line already exhibited abnormalities during the tetrad period. Using the tetrad period as the boundary, buds between 1 and 1.5 mm were selected to represent the early stage of pollen development, and buds ranging from 3.5 to 5 mm in length were taken to represent the late stage of pollen development for transcriptome sequencing. The sequencing results averaged 42, 154, 243;41, 243, 279; 41, 333, 469, and 41, 328, and 565, respectively. There were 74.59Gb clean reads in total, and 85.76%, 86.67%, 88.03%, and 87.86% of the reads were successfully mapped to the reference genomes of E-366-2S, E-366-2F, L-366-2S, and L-366-2F, respectively. The groups were screened for DEGs using |fold change| ≥ 2 and *p* < 0.01 as criteria. A total of 6982 DEGs were found between 366 and 2S and 366-2F during the early period, 13,224 DEGs were detected during the late period, and 2583 DEGs were common in both periods (Figure S1). Among the early DEGs, 2927 and 4055 genes were downregulated and upregulated, respectively; in the late period, 4599 and 8625 genes were downregulated and upregulated, respectively (Figure S2). The target gene *BraA09g012710.3C* was barely expressed in 366-2S in the early stage, highly expressed in 366-2F, and barely expressed in both fertile and sterile in the late stage (Figure 5B).

### 2.12. KEGG and GO Enrichment Analyses of DEGs

**Figure 7:**
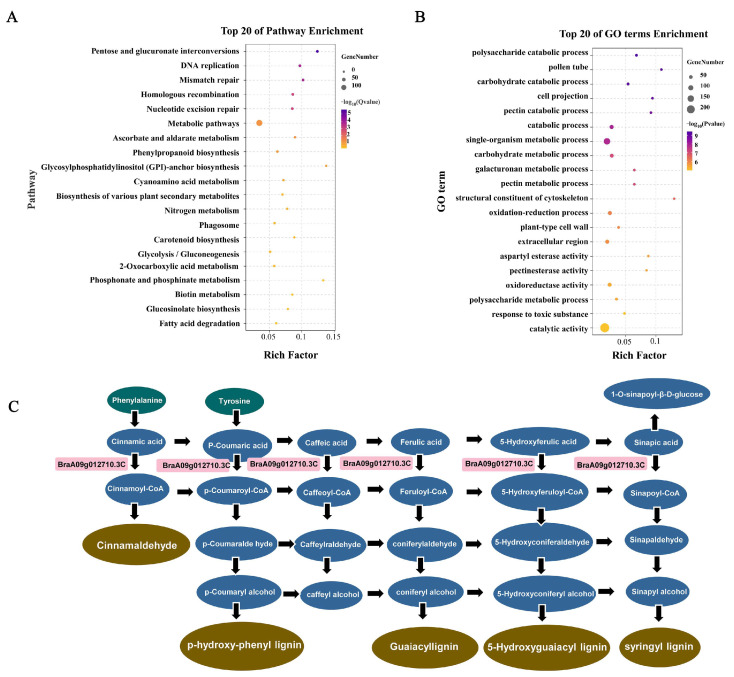
*BraA09g012710.3C* enrichment analysis and mapping of the metabolic pathway in which it is located. (**A**) Bubble map of Kyoto Encyclopedia of Genes and Genomes (KEGG) enrichment of differentially expressed genes (DEGs). (**B**) Bubble map of Gene Ontology (GO) enrichment of DEGs. (**C**) Phenylpropane metabolic pathway map.

To investigate the main pathways and functions in which the DEGs exerted their influence, KEGG and GO enrichment analyses were performed on the screened DEGs using |fold change| ≥ 10 and *p* < 0.01 as criteria. The results of KEGG enrichment analysis revealed that the DEGs were significantly enriched in the pathways of pentose and glucuronic acid interconversion, aldolate metabolism, and phenylpropane biosynthesis (Figure 7A). Among them, *BraA09g012710.3C* was enriched in the phenylpropane biosynthesis pathway. GO enrichment analysis was conducted to assign DEGs to three functional categories to elucidate the differences in the gene function of DEGs between samples, and bubble plots were created to illustrate the functions of the top 20 significantly enriched genes. The results showed that at the cellular component level, DEGs were significantly enriched in pollen tubes and cell walls; in terms of molecular functions, the DEGs were significantly enriched in oxidoreductase activity and pectinase activity; and in the biological process category, they were significantly enriched in polysaccharide catabolism metabolism, pectin metabolism, and single-organism metabolism (Figure 7B). Among these, the majority of mRNAs encoded pectin esterases and polygalacturonases, both of which are pectinases that regulate pectin degradation in the cell wall. There were also many mRNAs encoding glycosidases, such as galactosidases and glucosidases, which can participate in the hydrolysis of polysaccharides in the cell wall. Moreover, most of these DEGs exhibited a downregulation pattern in 366-2S, indicating that the expression of most enzymes related to cell wall degradation was downregulated, which may lead to delayed degradation of the tapetum [25].

### 2.13. Defects in the Phenylpropane Metabolic Pathway Affect Sporopollenin Synthesis

The above results demonstrated that *BraA09g012710.3C* was deleted in 366-2S and that its early stage of mRNA expression was significantly different between 366 and 2S and 366-2F. In addition, the gene was highly expressed in flowers and buds and was hypothesized to be a key gene causing sterility in 366-2S. The KEGG enrichment results showed that *BraA09g012710.3C* was enriched in the phenylpropanoid metabolic pathway and that its deletion may have caused an abnormality in the phenylpropanoid metabolic pathway. The phenylpropanoid metabolic pathway occurs in the tapetum layer, which is the site of sporopollenin synthesis [27]. The phenylpropanoid metabolic pathway produces numerous phenylpropanoid derivatives, including p-hydroxybenzoate (p-BA), p-coumarate (p-CA), ferulic acid ester (FA), and lignin guaiacyl (G) (Figure 7C). Research has shown that mutants with an abnormal phenylpropanoid metabolic pathway do not form sporopollenin normally and that phenylpropanoid derivatives are important constituents of sporopollenin [28]. It is hypothesized that the abnormality of the phenylpropane metabolic pathway affects the development of the tapetum layer. Due to this interference, the tapetum layer is unable to synthesize sporopollenin precursors, cannot provide nutrients for pollen development, and cannot undergo apoptosis at a later stage. This ultimately leads to the failure to form sporopollenin, which is a major constituent of the outer walls of the anther, resulting in the loss of the outer wall of the anther. In addition, the tapetum layer is too thick resulting in the inability to form individual microspores and ultimately male sterility due to the inability to form pollen.

## 3. Materials and Methods

### 3.1. Plant Materials and Growth Conditions

The genic male-sterile mutant line 366-2S was derived from Chinese cabbage breeding material 366-2, in which the progeny was segregated into fertile and sterile types at a ratio of 3:1 during reproduction. The corresponding homozygous fertile line, identified as the wild type (366-2F line), and the sterile DH line Y636-9 of Chinese cabbage were selected as the male and female parents, respectively, to generate an F_2_ segregation population for gene fine mapping. Eighteen DH lines were crossed with 366-2S, resulting in infertile offspring, which confirmed 366-2S as a recessive single-gene mutation (Table S2). All plant materials were cultivated in the standard experimental field of the Henan Modern Agriculture Research and Development Base, Yuanyang, Henan Province, China.

### 3.2. Alexander Staining and DAPI Staining

During the flowering period, inflorescences were collected and fixed in Carnoy’s solution (ethanol: acetic acid = 3:1) for at least 4 h at room temperature (25 °C). The fixed materials were subsequently dehydrated in 90% and 70% ethanol for 30 min each and finally stored in 70% ethanol at 0–4 °C. Pollen grains from the 366-2S (sterile) and 366-2F (fertile) lines were subjected to Alexander staining [29]. The anther development was observed under a Nikon ECLIPSE 80i microscope (Nikon, Tokyo, Japan), and micrographs were captured. The microspore development in the 366-2S and 366-2F lines was visualized using DAPI staining [30]. Prepared slides were examined under a fluorescence microscope (Olympus BX43, Tokyo, Japan), and photomicrographs were captured for documentation.

### 3.3. Anthers Sectioned in Paraffin

To identify the pollen abortion period, flower buds from the 366-2S and 366-2F lines were fixed in FAA solution (50% ethanol, 5% glacial acetic acid, and 10% formalin) and then dehydrated in a graded ethanol series. Subsequently, the prepared anthers were embedded in paraffin, stained with hematoxylin and eosin (HE), and observed under an optical microscope (Nikon ECLIPSE 80i; Nikon, Tokyo, Japan) [31]. Buds were categorized into five grades based on their lengths: BUD1 (<1 mm), BUD2 (1–1.5 mm), BUD3 (1.5–2.5 mm), BUD4 (2.5–3.5 mm), and BUD5 (>3.5 mm).

### 3.4. Scanning Electron Microscopy Observation of Mature Anthers

At the full-bloom stage, mature anthers were collected from both sterile and fertile plants and fixed in a 2.5% glutaraldehyde solution at 4 °C for 24 h. The samples were dehydrated in a series of graded ethanol solutions (30%, 50%, 70%, 85%, 95%, 100%, and 100%, each for 15 min). The samples were subsequently immersed in a mixture of isoamyl acetate and ethanol (1:1) for 10 min, followed by immersion in pure isoamyl acetate for an additional 10 min. After dehydration, the samples were dried using a critical point dryer (Quorum K850; Quorum, East Sussex, UK) and then sputter-coated with gold using an ion sputtering instrument (HITACHI MC1000; HITACHI, Tokyo, Japan). Scanning electron microscopy (SEM) (HITACHI Regulus 8100; HITACHI, Japan) was used to conduct observations [32].

### 3.5. BSA Sequencing Database Construction and Data Analysis

Based on the fertility identification results for individual plants in the F_2_ population, 30 fertile and 30 sterile plants were selected to establish the fertile pool (366F) and sterile pool (366S), respectively. The total DNA was extracted from each sample using the CTAB method, including DNA extraction from the fertile parent Y636-9 and sterile parent 366-2 [33]. Jinosec Technology Co., Ltd. (Wuhan, China) conducted paired-end 150 bp (PE150) sequencing on the Illumina HiSeq platform (San Diego, CA, USA). Clean reads were aligned to the *B. rapa* reference genome (V3. 0) [26] by using the BWA software package (v0.7.17) to obtain SAM files, which were subsequently converted into BAM files with Samtools [34]. SNP variations were detected using GATK [35], and SNP index analysis was performed using QTLeqr software [36] with Chiifu Chinese cabbage as the reference. The SNP index distributions were mapped to chromosomes with a 2 Mb sliding window, and the average SNP index value within each window was calculated. Regions above the 95% threshold line were identified as candidate intervals for the sterility gene.

### 3.6. KASP Marker Development and Genetic Mapping

The KASP method was used to conduct an initial linkage analysis of the target gene. First, SNP markers that exhibited polymorphism between the two DNA libraries and were located near the candidate BSA sequencing (BSA-seq) region were selected for KASP marker development. The F_2_ population, consisting of 325 individuals, was then genotyped using KASP markers (Table S10) that exhibited polymorphism between the parental lines. A genetic linkage map was constructed using JoinMap 4.0 software.

The F_2_ segregating population was expanded, resulting in the selection of 708 sterile plants. Flanking markers within the preliminary candidate interval were utilized for genotyping, and recombinant plants were identified. KASP primers were designed for the preliminary candidate interval, and wild-type Y636-9, mutant 366-2S, and F_1_ plants (as controls) were subjected to KASP marker polymorphism screening. Subsequently, polymorphic KASP markers were used to genotype the selected recombinant plants and both parental lines, further narrowing down the candidate interval. Genes within the candidate interval underwent functional annotation, sequence variation analysis, and gene expression analysis to identify potential candidate genes involved in male sterility.

### 3.7. Gene Cloning and Sequence Analysis

Full-length primers for *BraA09g012710.3C*, along with upstream and downstream primers for the 366-2S deletion, were designed based on the gene analysis results and Integrative Genomics Viewer data (Table S5). The candidate gene *BraA09g012710.3C* was cloned using the Phanta^®^ High-Fidelity Enzyme Mix (Vazyme, Nanjing, China) in a total reaction volume of 50 µL, comprising 3 µL of DNA template, 3 µL of both forward and reverse primers, 25 µL of enzyme mix, and 16 µL of ddH2O. The polymerase chain reaction (PCR) conditions followed the manufacturer’s instructions. The PCR products were sequenced by Sunya Biotech Co., Ltd. (Zhengzhou, China). Sequence alignment of 366-2S and Y636-9 was conducted using DNAMAN.

### 3.8. Design of Molecular Markers Based on Candidate Genes

Based on the sequence differences in the *BrACOS5* candidate genes between the 366-2S and Y636-9 parents, primers were designed and verified in 22 F_2_ populations (Table S5). The PCR amplification program consisted of initial denaturation at 95 °C for 5 min, followed by 32 cycles at 95 °C for 30 s, 60 °C for 30 s, and 72 °C for 20 s, with a final extension at 72 °C for 5 min. The PCR products were analyzed using 1.5% agarose gel electrophoresis.

### 3.9. RNA Extraction and Quantitative Real-Time PCR Analysis

At the flowering stage, roots, stems, leaves, flowers, buds, and other tissues were collected from 366 to 2S and 366-2F, flash-frozen in liquid nitrogen, and stored at −80 °C. The total RNA was extracted from all samples using the TransScript One-Step gDNA Removal and cDNA Synthesis Kit (Trans, Beijing, China) following the manufacturer’s instructions and reverse-transcribed to obtain cDNA. Quantitative primers for the candidate gene *BraA09g012710.3C* were designed online (https://www.ncbi.nlm.nih.gov/, accessed on 20 July 2024) (Table S9). The expression of *BraA09g012710.3C* in various tissues was assessed using quantitative real-time PCR (qPCR). qPCR was conducted using the Roche Light Cycler 480-II system (Roche Applied Sciences, Beijing, China), with the *BrGAPDH* gene serving as an internal reference gene, using three replicates. The relative expression levels were calculated using the 2−ΔΔCT method [37]. The amplification procedure for qPCR consisted of pre-denaturation at 95 °C for 30 s, followed by 45 cycles of the following reactions: 95 °C for 5 s, 60 °C for 20 s, and 72 °C for 20 s, followed by melt curve analysis: 95 °C for 5 s, 60 °C for 1 min, and finally cooling at 50 °C for 30 s. The reaction system consisted of 1 µL of cDNA, 5 µL of TB Green Premix Ex Taq II (2×), 0.4 µL of Feverse primer, 0.4 µL of Reverse primer, and 3.2 µL of RNase-Free Water.

### 3.10. Validation of the Expression of BraA09g012720.3C, BraA09g012730.3C and BraA09g012740.3C in F_2_ Sterile Populations

Since four genes were included in the 27 kb deletion, we designed primers (Table S6) for the other three genes and verified the expression of these three genes in the sterile F2 population by PCR amplification. The PCR amplification program consisted of initial denaturation at 95 °C for 5 min, followed by 35 cycles at 95 °C for 30 s, 58 °C for 30 s, and 72 °C for 1 min, with a final extension at 72 °C for 5 min. The PCR products were analyzed using 1.5% agarose gel electrophoresis.

### 3.11. Phylogenetic Analysis

The *BraA09g012710.3C* gene, annotated as *BrACOS5*, encodes acyl-CoA synthetase 5. The *BrACOS5* protein sequences were compared in the GenBank database using BLAST (https://www.ncbi.nlm.nih.gov/genbank/, accessed on 28 July 2024), and homologous sequences of maize, rice, Arabidopsis thaliana, oilseed rape, and Chinese cabbage were downloaded. We also used MEGA12 for sequence comparison and phylogenetic tree construction, the clustal W algorithm to compare the amino acid sequences, and the maximum likelihood method for phylogenetic reconstruction, the bootstrap validation parameter is 1000 [38,39,40,41].

### 3.12. Transcriptome Sequencing and Differential Expression Analysis

Cytological observations revealed abnormalities in 366-2S from the tetrad period onwards. Based on this observation, the stages before and after the tetrad period were designated as the early pollen (E) and late pollen (L) stages, respectively. Buds measuring between 1 and 1.5 mm were selected as samples representing early pollen development, while buds ranging from 3.5 to 5 mm in length represented late pollen development. A total of 12 mRNA sequencing samples were collected, comprising four treatments (E-366-2S, E-366-2F, L-366-2S, and L-366-2F) with three replicates per treatment.

The mRNA (coding sequences) from the total RNA was enriched using Oligo dT magnetic beads, followed by the construction of sequencing libraries for all samples. Subsequently, 12 cDNA libraries were sequenced on the Illumina HiSeq 2500 platform (150 bp paired-end sequencing; 6 Gb data size). Clean reads were obtained for all treatments and aligned to the *B. rapa* reference genome (v3. 0). Reads were assembled into annotated mRNA and unannotated mRNA (potentially representing novel genes) based on known genome annotations. The fragments per kilobase of transcript per million mapped reads (FPKM) values were calculated for all assembled mRNA in each sample. Correlation analysis was performed on three replicates for each treatment to assess reproducibility, with the average used as the expression profile for each treatment [25].

Differential expression analysis of mRNA profiles was conducted between the early group (E-366-2S vs. E-366-2F) and the late group (L-366-2S vs. L-366-2F). Differentially expressed genes (DEGs) were identified using |fold change| ≥ 2 and *p* < 0.01 as criteria.

### 3.13. Kyoto Encyclopedia of Genes and Genomes and Gene Ontology Enrichment Analyses

Functional annotation was performed by comparing the DEG sequences with the non-redundant (Nr), BLAST search [42], Swissprot [43], Kyoto Encyclopedia of Genes and Genomes (KEGG) [44], and Gene Ontology (GO) databases [45]. Using |log2fc| > 10 as a criterion, DEGs were selected for further enrichment analysis using KEGG and GO terms. Enrichment analysis and visualization were conducted using the Kidio cloud platform (https://www.omicshare.com/, accessed on 3 August 2024).

## 4. Discussion

Chinese cabbage is a typical cross-pollinated crop that shows strong heterosis and high hybrid vigor [17]. Today, the production of hybrid varieties is an ideal breeding goal [46]. An effective and stable pollination control mechanism is essential for hybrid seed production [47,48]. Most traditional breeding approaches adopt the artificial emasculation method to produce hybrid seeds, which increases the cost of seed production [49]. In contrast, plant male sterility is an ideal agronomic trait that provides a low-cost and high-efficiency method for variety improvement and hybrid seed production. Male-sterile lines are also useful in identifying genes related to anther and pollen development [50].

Starting from the formation of sporogenous cells, pollen mother cells are produced through mitosis, and the pollen mother cells generate tetrads via meiosis. As the callose wall degrades, the tetrads separate, releasing individual microspores that gradually develop into pollen grains [51]. Abnormal development of these processes can lead to pollen sterility or complete absence of pollen grains [52]. In this study, we isolated a male-sterile mutant line 366-2S with a specific genotype from the Chinese cabbage breeding material 366-2 and identified the corresponding homozygous fertile line 366-2F. By observing DAPI-stained spores at different stages in 366-2S and 366-2F and examining anther development through HE staining, we found that anther development in 366-2F was normal, while in 366-2S, the tapetum layer degraded late during the binucleate and trinucleate stages, causing microspores to aggregate and fail to separate, ultimately failing to form viable pollen. The tapetum layer plays a crucial role in pollen development, and timely deposition and degradation of the tapetum layer are essential for normal pollen growth and development. Both premature and delayed degradation of the tapetum layer can lead to pollen sterility, resulting in male sterility in plants [53,54,55].

In this study, we used BSA-seq to map the gene associated with pollen sterility to a 65 kb physical interval containing 11 genes. Through gene annotation and functional analysis, we identified *BrACOS5* (*BraA09g012710.3C*) as being related to lipid metabolism. Previous studies have shown that *ACOS5* is essential for pollen biosynthesis and development, *ACOS5* is primarily expressed in the tapetum layer of the anther, which plays a vital role in the anther by providing nutrients, proteins, lipids, and other substances for microspore development [56,57]. The mutation of *ACOS5* leads to abnormal development of the tapetum layer, preventing the formation of microspores and resulting in male sterility in *Arabidopsis thaliana* [58]. Similar findings have been reported in maize, where *ZmACOS5* is closely related to pollen development [59]. *OsACOS12*, the ortholog of *AtACOS5*, also plays a significant role in rice pollen development [60]. Through phylogenetic analysis, we found that *BrACOS5* in Chinese cabbage shares a high homology with *AtACOS5*, suggesting that *BrACOS5* is likely closely related to pollen development as well. To further confirm the role of *BrACOS5* in pollen development, we sequenced 366-2S and 366-2F and found a 27 kb deletion in 366-2S, including the *BrACOS5* gene. Regarding the mutations in *BrACOS5*, Zou et al. identified three allelic male-sterile mutants (msm2-1/2/3) in the progeny of Chinese cabbage induced by EMS (Ethylmethanesulfonate). These male-sterile phenotypes were caused by three different SNP mutations in *BrACOS5* [24]. Compared to their mutations, the 27 kb deletion we discovered, which includes BrACOS5, has not been reported by previous researchers and represents a specific mutation. Quantitative PCR (qPCR) analysis revealed that *BrACOS5* is almost not expressed in 366-2S but is expressed in 366-2F, particularly at high levels in the buds. These results further support the hypothesis that the deletion of *BrACOS5* likely leads to abnormal pollen development.

It was reported that the separation of the tetrad relies on β-1,3-glucanase, which hydrolyzes the callose wall of the tetrad to facilitate the release of microspores [61]. Many studies have shown that β-1,3-glucanase is secreted by tapetal cells [62,63,64,65]. Abnormal development of the tapetum layer may affect the secretion of β-1,3-glucanase, which may not be able to degrade the callose layer in time and lead to failure of tetrad isolation. In the bnms3 mutant, delayed degradation of callose results in failure to release microspores [66]. By DAPI staining, we found that during the tetrad period, the microspores were suspected to be tetrad aggregates or pollen mother cells that were not separated, If the tetrads are gathered in clusters, it may be related to untimely degeneration of the callose layer.

Through KEGG annotation of *BrACOS5*, we found that *BrACOS5* is mainly involved in the phenylpropanoid metabolic pathway, which has been shown to be closely related to tapetum layer and sporopollenin synthesis, derivatives of the phenylpropanoid metabolic pathway are essential for sporopollenin synthesis [28]. The absence of *BrACOS5* may lead to abnormalities in the phenylpropanoid metabolic pathway, thereby affecting the synthesis of sporopollenin. The GO enrichment analysis showed significant enrichment in carbohydrate metabolism and cell wall-related processes. The mRNAs involved in these significantly enriched GO terms mainly encoded pectinesterase, polygalacturonase, galactosidase, and glucosidase, which are primarily involved in cell wall degradation. In the sterile line 366-2S, most of these mRNAs exhibited downregulated expression, which may inhibit cell wall degradation during certain developmental processes. Moreover, our observations from paraffin sectioning revealed delayed degradation of the tapetum layer in the sterile line 366-2S. Therefore, we propose that the downregulation of these cell wall degradation-related mRNAs in the sterile line 366-2S may hinder the degradation of the tapetum layer.

In summary, the downregulation of mRNAs related to cell wall degradation in 366-2S may impede the degradation of the tapetum layer. Moreover, the absence of *BrACOS5* leads to abnormalities in the phenylpropanoid metabolic pathway and tapetum layer development, affecting the synthesis of sporopollenin. The delayed degradation of the tapetum layer results in the failure of tetrad separation and the inability to release viable individual microspores, thereby causing male sterility.

## 5. Conclusions

In this study, the newly discovered male-sterile natural mutant Chinese cabbage 366-2S was used as the experimental material, and analyses were conducted to locate the sterile gene. In the early stage, the results confirmed that the sterility trait was a single-gene recessive inheritance based on the construction of an F_2_ segregating population. The sterile gene was located on chromosome A09 and was about 65 kb. *BraA09g012710.3C* may be a candidate gene for the sterile trait in 366-2S. In 366-2S, the deletion of *BraA09g012710.3C* affected the pathways related to sporopollenin biosynthesis and eventually led to male sterility. This study lays a foundation for the gene cloning and molecular mechanism analysis of Chinese cabbage nuclear male sterility and provides materials and theoretical support for the application of Chinese cabbage hybrid breeding.

## Data Availability

The original contributions presented in the study are publicly available. These data can be found in the China National GeneBank DataBase (CNGBdb) under accession number CNP0004830.

## Supplementary Materials

The following supporting information can be downloaded at: https://www.mdpi.com/article/10.3390/plants14050779/s1, Figure S1: Venn diagram of early and late DEGs. Figure S2. Histograms of up- and down-regulation of early and late DEGs. Table S1: Genetic analysis of 366-2S sterility characters. Table S2: Results of 366-2S crossed with other different genetic background Double Haploid lines. Table S3: Quality control analysis of raw data for BSA-seq. Table S4: Functional annotation of genes in candidate regions. Table S5: *BraA09g012710.3C* sequence analysis primer. Table S6: *BraA09g012720.3C*, *BraA09g012730.3C*, and *BraA09g012740.3C* sequence amplification primer. Table S7: Proportion of the three deleted genes present in the F_2_ sterile population. Table S8: FPKM values for *BraA09g012720.3C*, *BraA09g012730.3C*, and *BraA09g012740.3C*. Table S9: *BraA09g012710.3C* specific quantitative primer. Table S10: Gene location primers for sterility. Table S11: Sequence ID of amino acid sequences used in the phylogenetic tree.
