# Supplementary material for: Genetic Analysis and Fine Mapping of Spontaneously Mutated Male Sterility Gene in Chinese Cabbage (Brassica rapa L. ssp. pekinensis)
Source: Plants (Basel). 2025 Mar 3;14(5):779. doi: 10.3390/plants14050779 (PMC11902244; doi:10.3390/plants14050779)
Supplement: Supplementary file 1 [file plants-14-00779-s001.zip › Supplementary Figures.pdf]

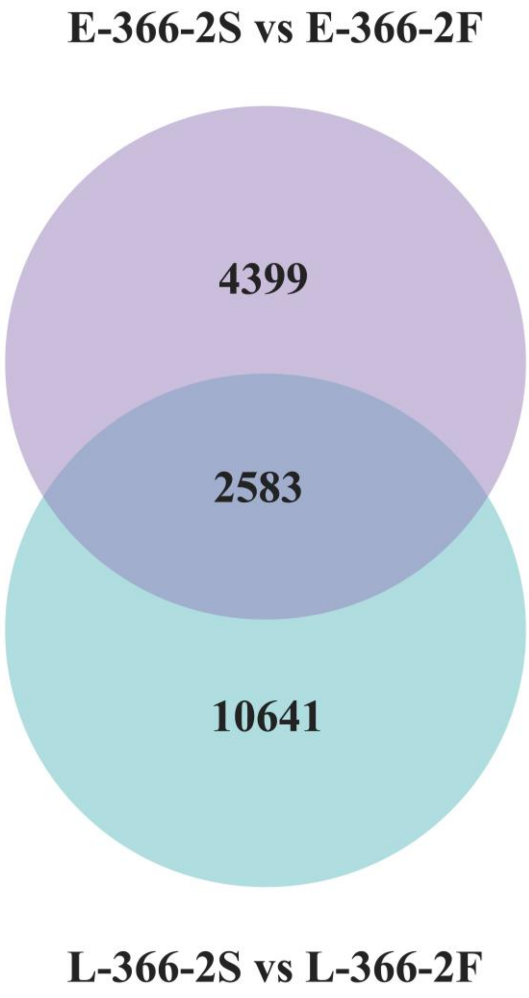

Figure S1. Venn diagram of early and late DEGs

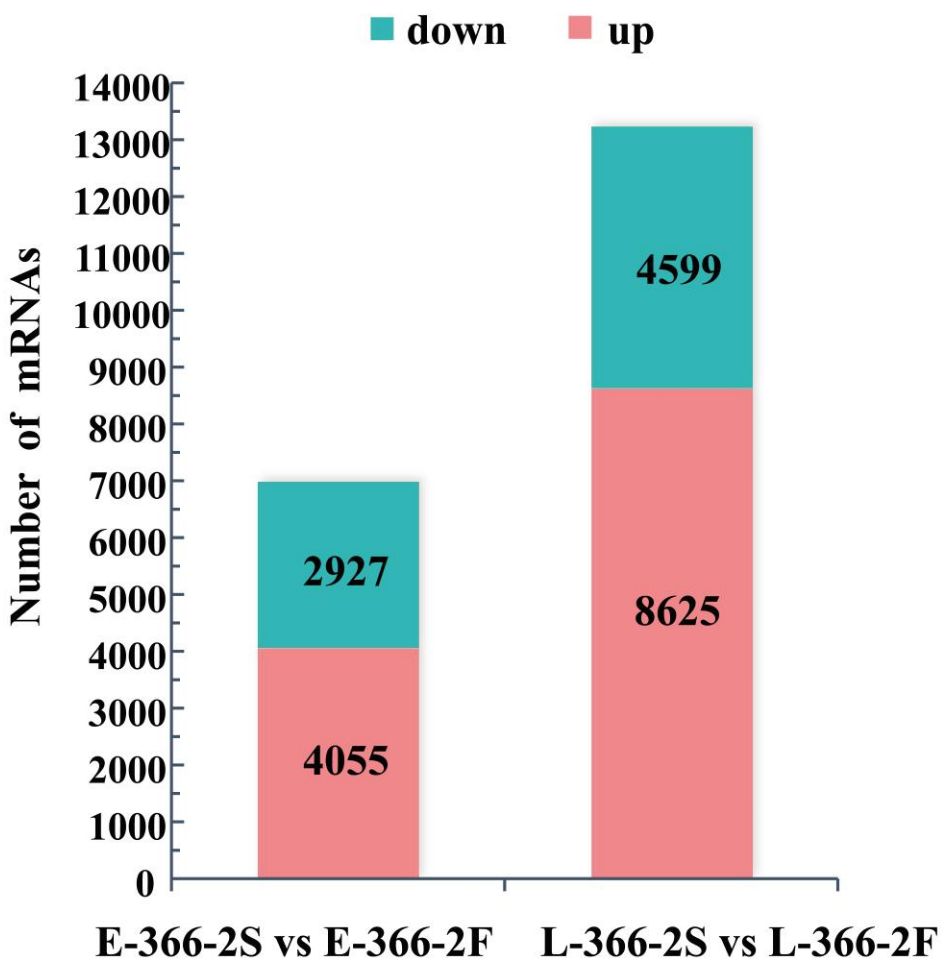

Figure S2. Histograms of up- and down-regulation of early and late DEGs
